# Differential associations of proinflammatory and anti-inflammatory cytokines with depression severity from noncancer status to breast cancer course and subsequent chemotherapy

**DOI:** 10.1186/s12885-020-07181-w

**Published:** 2020-07-23

**Authors:** Bor-Show Tzang, Vincent Chin-Hung Chen, Ching-Chuan Hsieh, Wen-Ke Wang, Yi-Ping Weng, Hsing-Ying Ho, Ya-Ting Hsu, Han-Pin Hsaio, Jun-Cheng Weng, Yi-Lung Chen

**Affiliations:** 1 Institute of Biochemistry, Microbiology and Immunology, Chung Shan Medical University, Taichung, Taiwan; 2 Department of Biochemistry, School of Medicine, Chung Shan Medical University, Taichung, Taiwan; 3 Clinical Laboratory, Chung Shan Medical University Hospital, Taichung, Taiwan; 4 Department of Psychiatry, Chang Gung Medical Foundation, Chiayi Chang Gung Memorial Hospital, Chiayi, Taiwan; 5 School of Medicine, Chang Gung University, Taoyuan, Taiwan; 6 Graduate Institute of Clinical Medical Sciences, College of Medicine, Chang-Gung University, Taoyuan, Taiwan; 7 Department of Surgery, Chang-Gung Memorial Hospital, Taoyuan, Taiwan; 8 Department of Surgery, Taipei Medical University Hospital, Taipei, Taiwan; 9 Breast center, Chiayi Chang Gung Memorial Hospital and University, Chiayi, Taiwan; 10 Department of Medical Imaging and Radiological Sciences, Chang Gung University, Taoyuan, Taiwan; 11 Department of Healthcare Administration, Asia University, 500, Lioufeng Rd., Wufeng, Taichung, 41354 Taiwan; 12 Department of Psychology, Asia University, Taichung, Taiwan

**Keywords:** Cytokines, Depression, Breast cancer, Chemotherapy, Moderation

## Abstract

**Background:**

In this study, we examined the differential associations of various proinflammatory and anti-inflammatory cytokines with depression severity from the development of breast cancer to subsequent chemotherapy treatment.

**Methods:**

A cross-sectional study was conducted on a sample of 116 women: 29 controls without cancer, 55 patients with breast cancer who were not receiving chemotherapy, and 32 patients with breast cancer who were receiving chemotherapy. Blood samples were assayed to evaluate serum levels of the following cytokines: interferon-γ, interleukin (IL)-12 (p70), IL-1β, IL-2, tumor necrosis factor (TNF)-α, IL-4, IL-5, IL-10, IL-13, IL-6, and IL-17A. Depression severity was assessed using the Patient Health Questionnaire.

**Results:**

After adjustment for sociodemographics, consistent patterns of the association between cytokine and depression were noted in the different groups. No significant associations were observed in the controls. Inverse associations were observed between cytokines levels and depression severity in patients with breast cancer who were not receiving chemotherapy, whereas positive associations were noted in patients with breast cancer who were receiving chemotherapy. Specific differential relationships between IL-5 levels and depression severity were found between patients with breast cancer who were receiving and not receiving chemotherapy.

**Conclusions:**

Our study revealed differential relationships between cytokine levels and depression severity with the development of cancer. Immunostimulation and immunosuppression in breast cancer and cancer treatment may account for the differential responses with the development of breast cancer.

## Background

Breast cancer is the most common invasive cancer affecting women and the leading cause of cancer-related morbidity and mortality among women worldwide [1]. The triggers for breast cancer are diverse, including hereditary and environmental factors [2]. Recently, cancer has been believed to result from inflammation; inflammation is considered to be associated with tumor development through the induction of cytokines—the secretory proteins that play crucial roles in intercellular communication [3]. Several studies have indicated that cytokines are the major regulators in the development of breast cancer and have demonstrated how they affect tumor cell behavior or reprogram the tumor niche through specific signaling pathways [4]. Studies have reported the existence of interleukin (IL)-1 family, IL-6, IL-11, IL-18, and interferons (IFNs) within tumor microenvironments and in metastatic sites [5]. Some of these cytokines, such as IL-1, IL-6, IL-11, and transforming growth factor (TGF)-β, stimulate breast cancer proliferation and invasion, whereas other cytokines such as IL-12p70, IL-18, and IFNs exert opposite effects on breast cancer proliferation or invasion [6]. In addition, the IL-17 family was found to be associated with poor prognosis of breast cancer [7]. Upregulated T helper 17 cells are positively correlated with IL-17 and are associated with tumor aggressiveness through the induction of angiogenic factors in patients with breast cancer.

Notably, breast cancer survivors are more likely than the general population to develop depression [8]; several studies that have evaluated the role of proinflammatory cytokines (such as IL-6 and IFNγ) in the development of depression in patients with breast cancer have found positive relationships between proinflammatory cytokines and depression [9–17]. By contrast, some studies have not reported such correlations [18–20], and other studies have reported inverse relationships between cytokines and depression or negative mood in patients with breast cancer [21, 22]. Such inconsistent findings may be attributed to uncontrolled effect modifiers. In addition, in the aforementioned studies, the researchers typically only examined one or specific cytokines, and the associations of all the cytokines with depression in patients with breast cancer have not been studied thoroughly and simultaneously.

Chemotherapy is the conventional treatment for breast cancer, and it affects the immune system of the treated patients. Notably, behavioral problems and cytokines have been reported to increase within 3 months of primary treatment for early-stage breast cancer [19, 20]; however, the patients gradually recover in 6–12 months [18]. Further, chemotherapy in patients with breast cancer modifies the prediction capability of kynurenine concentrations for depression, which differ before and after chemotherapy [18]. Notably, kynurenine is the primary metabolic route of tryptophan catabolism and is considered to be highly regulated by cytokines [23]. Therefore, chemotherapy in patients with breast cancer may modify the role of cytokines in depression, which could be a possible explanation for the aforementioned inconsistent findings.

Therefore, we conducted a cross-sectional study to investigate the roles of proinflammatory and anti-inflammatory cytokines in depression severity and their associated changes from the noncancer status to breast cancer course and subsequent chemotherapy treatment. The study comprised three groups: (1) controls without cancer, (2) patients with breast cancer who were not receiving chemotherapy, and (3) patients with breast cancer who were receiving chemotherapy. Based on the inflammation theory of cancer and the inconsistent findings between inflammation-related cytokines and depression, we examined a series of proinflammatory and anti-inflammatory cytokines. The targeted proinflammatory cytokines were IL-1β, IL-2, IL-6, IL-12p70, IL-17A, IFNγ, and tumor necrosis factor (TNF)-α, whereas the anti-inflammatory cytokines were IL-4, IL-5, IL-10, and IL-13. We hypothesized that chemotherapy in patients with breast cancer might modify the associations between depression severity, and proinflammatory and anti-inflammatory cytokines.

## Methods

### Participants

This cross-sectional study was conducted on a sample of 116 women aged ≥20 (range, 24–79) years who were treated at the oncology clinic of Chiayi Chang Gung Memorial Hospital from November 2017 to February 2019. Eligible participants were those diagnosed as having breast cancer (stages 0–3) without evidence of metastasis. The exclusion criteria for participants in this study were as follows: (1) any neurological disorder or a lifetime history of severe head trauma; (2) history of mental retardation, bipolar disorder, schizophrenia, or substance-related disorders or suicide attempt within the 12 months preceding the study; (3) illiteracy; (4) history of developmental delay; (5) severe visual impairment (e.g., cataract and glaucoma); (6) current use of antidepressants; and (7) current pregnancy. Among all the recruited participants, 55 had not received chemotherapy, whereas 32 had received chemotherapy. Further, 29 age-matched controls without cancer were recruited from communities around Chiayi Chang Gung Memorial Hospital. All participants provided written informed consent before enrolment into the study. This study was approved by the Institutional Review Board of Chiayi Chang Gung Memorial Hospital (approval number: 201700252B0C603). After the acquisition of written informed consent, the participants were asked to complete questionnaires and proceed for serum collection. The serum collection for participants receiving chemotherapy was completed 3–6 months after completion of chemotherapy [24].

### Serum collection

Most blood samples were obtained during the daytime and few were obtained at night, with no fasting limitation. Whole blood (5 mL) was collected from each participant in Serum Blood Collection Tubes (BD Vacutainer; Becton Dickinson, Franklin Lakes, NJ, USA) without the use of any additive. The clotted blood samples were immediately centrifuged at 2000×*g* for 10 min, and the serum was then aliquoted and stored at − 70 °C.

### Serum cytokine assay

The MILLIPLEX® MAP Human High Sensitivity T Cell Magnetic Bead Panel 96-Well Plate Assay (Millipore Corp, Billerica, MA, USA) was used to determine the concentrations of cytokines (proinflammatory for IL-1β, IL-2, IL-6, IL-12p70, IL-17A, IFNγ, and TNFα; anti-inflammatory for IL-4, IL-5, IL-10, and IL-13), according to the manufacturer’s instructions. The intra-assay coefficients of variation (CVs) were lower than 6% for IL-12p70 and lower than 5% for other analytes. The inter-assay CVs were lower than 15% for IL-1β, IL-2, IL-4, IL-12, TNFα and lower than 20% for IL-5, IL-6, IL-10, IL-13, IL-17A, and IFNγ. The assay sensitivities (pg/mL) for cytokines were as follows: 0.14 for IL-1β, 0.18 for IL-2, 1.07 for IL-4, 0.10 for IL-5, 0.11 for IL-6, 0.51 for IL-10, 0.16 for IL-12p70 and TNFα, 0.24 for IL-13, 0.31 for IL-17A, and 0.47 for IFNγ.

### Depression severity

The nine-item version of the Patient Health Questionnaire (PHQ) was used to screen for depression severity over 2 weeks preceding the study based on a four-point scale, ranging from 0 (not at all) to 3 (nearly every day). The PHQ score ranged from 0 to 27, wherein a higher PHQ score indicated more severe depression. The rationale underlying the use of the PHQ was its high internal consistency, with a Cronbach’s alpha of 0.83 [25].

### Statistical analyses

Statistical analyses were conducted using SAS 9.4 (SAS Institute Inc., Cary, NC, USA). Descriptive results are presented as frequency and percentage for categorical variables and as mean and SD for continuous variables. Participants were categorized into three groups: (1) controls without cancer, (2) patients with breast cancer who were not receiving chemotherapy, and (3) patients with breast cancer who were receiving chemotherapy. The general linear model (GLM) was used to explore intergroup differences in sociodemographics (i.e., marital status, age, and year of education) and depression severity. In addition, owing to nonnormality in cytokines, the Kruskal–Wallis H test was used to explore intergroup differences of cytokines. Post hoc analysis with Bonferroni correction method was performed for intergroup comparisons if statistical significance was detected in the GLM or Kruskal–Wallis H test.

The GLM was used with various cytokines as predictors, depression severity as the outcome, and sociodemographics as covariates in each group to report the possible differential relationships of proinflammatory and anti-inflammatory cytokines in depression severity between the groups. Moderation analyses were applied to determine whether these effects of cytokines on depression severity significantly differed between the groups. In moderation analyses, two interaction terms were added in the GLM by using the total sample and the group of patients with breast cancer who were not receiving chemotherapy serving as the reference group. A statistically significant interaction was the one that indicated that the magnitude of regression coefficients (effects of cytokines on depression severity) differed between the reference (patients with breast cancer not receiving chemotherapy) and comparison groups (control group and patients with breast cancer receiving chemotherapy). Finally, to clarify whether some breast cancer-related factors (tumor mass, duration of completion of chemotherapy, and time since diagnosis of breast cancer) affect cytokine levels, we established a similar GLM with adjustment for sociodemographics in the group of patients with breast cancer who were receiving chemotherapy.

## Results

Sociodemographics, immune characteristics, and depression severity of the groups are presented in Table 1. All participants were female, and the majority of them were married (75.00–86.21%). Most of the participants in the cancer groups had stage II breast cancer at the time of diagnosis. The mean age and years of education of the participants ranged from 47.59 to 52.29 years and 10.56 to 12.79 years, respectively. In the group of patients with breast cancer who were not receiving chemotherapy, 78.18% of blood samples were obtained within 1 month after the patients were diagnosed as having breast cancer, and 70.37% of blood samples were collected before the patients underwent surgery for tumor removal. The average duration of completion of chemotherapy for patients who were receiving chemotherapy was 129.68 days; 71.88% of patients with breast cancer who received chemotherapy never received radiotherapy at the time of blood collection, whereas the remaining 28.13% of patients had received radiotherapy prior to chemotherapy.

**Table 1:** Sociodemographics, immune characteristics, and depression severity of study groups

Variable	Group			Statistics	Post-hoc^a^
	Noncancer control	Breast cancer group not receiving chemotherapy	Breast cancer group receiving chemotherapy		
	*N* = 29	*N* = 55	*N* = 32		
Marital status (married)	25 (86.21)	41 (74.55)	26 (81.25)	χ2 = 1.68; *p* = 0.433	–
Cancer stage					
0	–	5 (9.26)	0 (0)	–	–
1		15 (27.78)	9 (28.13)		
2		25 (46.30)	17 (53.13)		
3		8 (14.81)	6 (18.75)		
Age (years)	47.59 (11.09)	52.40 (11.06)	49.56 (10.72)	F = 1.96; *p* = 0.146	–
Education (years)	12.79 (3.70)	10.59 (4.37)	11.81 (2.95)	F = 3.26; *p* = 0.042*	1 > 2
Duration of completion of chemotherapy (days)	–	–	129.68 (50.57)	–	–
Type of chemotherapy					
Anthracyclines	–	–	29 (90.63)	–	–
Taxanes	–	–	22 (68.75)	–	–
Other DNA synthesis inhibitors	–	–	29 (90.63)	–	–
Other microtubule inhibitors	–	–	0 (0)	–	–
Tumor mass (cm^3^)	–	–	6.36 (9.68)		
Pro-inflammatory (pg/mL)					
IL-1β	0.97 (0.49)	0.91 (0.80)	0.70 (0.49)	H = 6.63; *p* = 0.036*	1 = 2 = 3
IL-2	1.70 (1.30)	1.53 (1.22)	0.96 (0.84)	H = 9.46; *p* = 0.009**	1 > 3
IL-6	2.41 (1.67)	1.83 (1.88)	1.72 (1.97)	H = 7.42; *p* = 0.025*	1 = 2 = 3
IL-12p70	3.35 (1.96)	2.57 (1.77)	2.01 (1.45)	H = 8.59; *p* = 0.014*	1 > 3
IL-17A	7.94 (3.78)	7.17 (3.73)	5.16 (2.52)	H = 11.90; *p* = 0.003**	1 = 2 > 3
IFNγ	12.83 (7.14)	8.71 (5.80)	7.26 (4.27)	H = 13.88; *p* = 0.001***	1 > 2 = 3
TNFα	5.74 (2.79)	5.41 (2.97)	5.17 (2.55)	H = 1.34; *p* = 0.513	–
Anti-inflammatory (pg/ml)					
IL-4	15.14 (10.10)	13.06 (11.58)	10.09 (7.16)	H = 4.54; *p* = 0.103	–
IL-5	1.73 (1.19)	1.80 (1.30)	1.25 (0.98)	H = 4.40; *p* = 0.111	–
IL-10	6.41 (4.86)	6.02 (4.75)	3.69 (3.46)	H = 8.02; *p* = 0.018*	1 = 2 = 3
IL-13	4.81 (2.67)	3.36 (2.89)	3.12 (4.19)	H = 12.66; *p* = 0.002**	1 = 2 = 3
PHQ (depression)	2.00 (2.58)	5.42 (4.72)	3.28 (2.67)	F = 8.54; *p* < 0.001***	2 > 1 = 3

Data are shown as N (%) for marital status and cancer stage, and mean (standard deviation) for the other variables

*IL* interleukin, *IFN* interferon, *PHQ* patient health questionnaire, *TNF* tumor necrosis factor

^a^group 1 = controls without cancer; group 2 = patients with breast cancer who were not receiving chemotherapy, group 3 = patients with breast cancer who were receiving chemotherapy

Bonferroni correction was used for post hoc comparisons

Data regarding cancer stage of two patients were missing in the group of patients with breast cancer who were not receiving chemotherapy

*p < 0.05, **p < 0.01, ***p < 0.001

Based on the univariate analysis of the GLM, we found significant differences in the years of education, cytokines (IL-1β, IL-2, IL-6, IL-12p70, IL-17A, IFNγ, TNFα, IL-10, and IL-13), and depression severity (indicated by the PHQ) among the groups. Further post hoc analysis with Bonferroni’s correction demonstrated that controls without cancer had one more year of education compared with patients with breast cancer who were not receiving chemotherapy. The levels of all cytokines were the highest in the controls, followed by patients with breast cancer who were not receiving chemotherapy; the lowest cytokine levels were found in patients with breast cancer who were receiving chemotherapy. Specifically, IFNγ levels were higher in noncancer controls than in patients with breast cancer, regardless of the chemotherapy status. The levels of cytokines IL-2 and IL-12p70 were higher in the controls than in patients with breast cancer who were receiving chemotherapy. In addition, patients with breast cancer who were receiving chemotherapy had the lowest levels of IL-17A, and no differences were observed between controls and patients with breast cancer who were not receiving chemotherapy. Finally, depression severity was higher in patients with breast cancer who were not receiving chemotherapy than in controls and patients with breast cancer who were receiving chemotherapy.

**Fig. 1:**
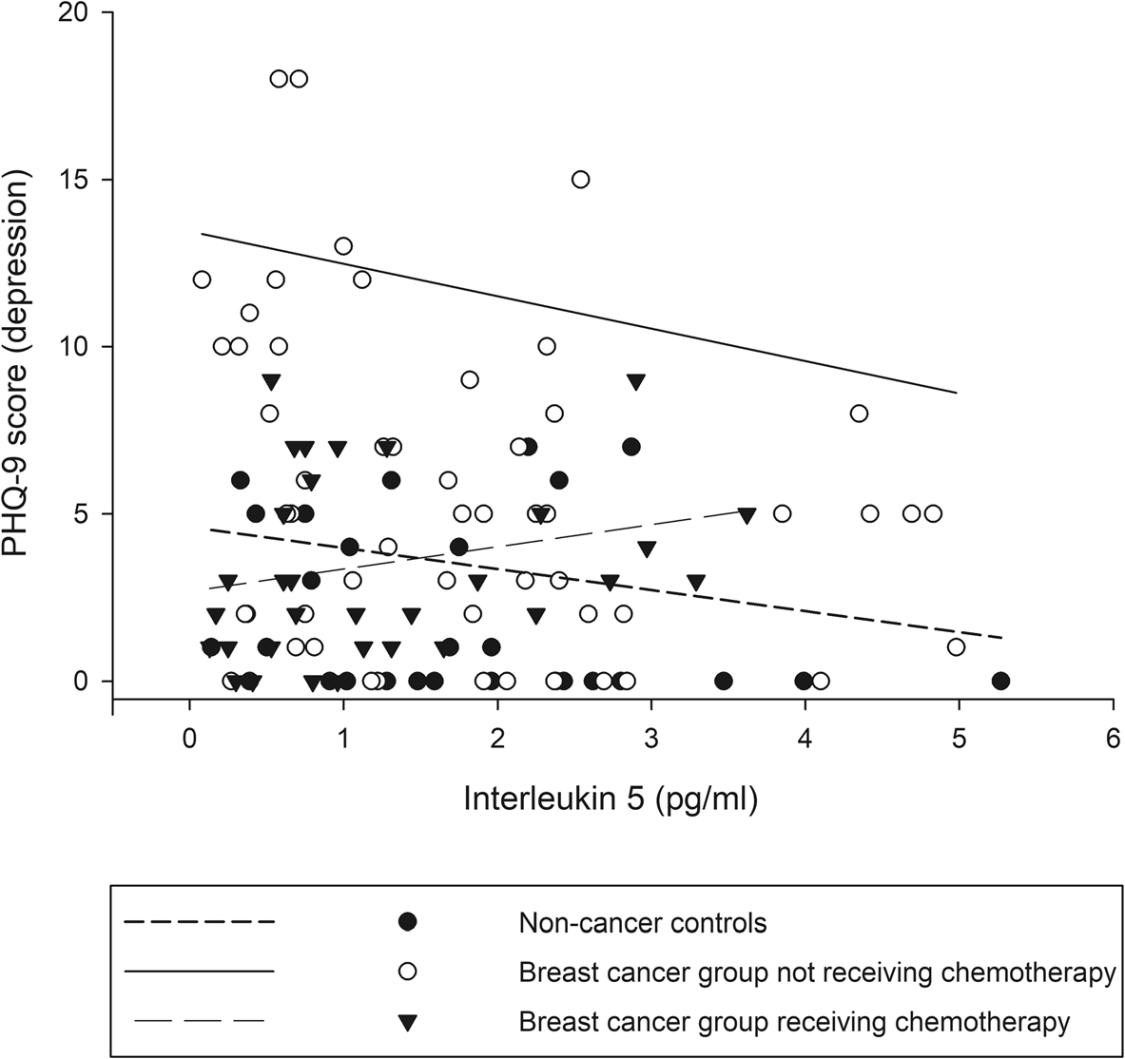
Differential effects of IL-5 levels on depression severity between controls, and patients with breast cancer receiving and not receiving chemotherapy. We used the linear regression analysis to visualize the effects of cytokines on depression severity after adjustment for sociodemographics between the groups. Although IL-5 level and depression severity was not significantly associated in different groups, based on the moderation analysis, the differential association between IL-5 levels and depression severity between breast cancer groups receiving (regression coefficient = 0.66) and not receiving chemotherapy (regression coefficient = − 0.97) was 1.59 of regression coefficient

Table 2 presents the results of the GLM established to examine the relationship between cytokine levels and depression severity in different groups, after adjustment for sociodemographics, and their interactions. In general, differential, consistent patterns of relationships between proinflammatory and anti-inflammatory cytokines and depression severity were observed between the groups. In the control group, no associations were found between proinflammatory and anti-inflammatory cytokines and depression severity, and most regression coefficients were small (i.e., less than 0.1), except for IL-1β and IL-5. In patients with breast cancer who were not receiving chemotherapy, inverse associations were observed between most proinflammatory and anti-inflammatory cytokines and depression severity, especially IL-2 (regression coefficient = − 1.20). Although positive associations were observed for some cytokines (i.e., TNFα and IL-4), their associations were minimal (i.e., 0.02 and 0.07). By contrast, positive relationships between cytokine levels and depression severity were noted in patients with breast cancer who were receiving chemotherapy, especially IL-12p70 (regression coefficient = 0.81), although many results did not reach statistical significance. Furthermore, differential relationships between IL-5 and depression severity in different groups were also reflected in moderation analyses. Although IL-5 level and depression severity was not significantly associated in different groups, based on the moderation analysis, the differential association between IL-5 levels and depression severity between breast cancer groups receiving (regression coefficient = 0.66) and not receiving chemotherapy (regression coefficient = − 0.97) was 1.59 of regression coefficient (Fig. 1).

**Table 2:** Differential effects of cytokines on depression in controls and patients with breast cancer receiving/not receiving chemotherapy

Cytokines	Effect of cytokines on depression between groups			Noncancer control vs. Breast cancer group not receiving chemotherapy	Noncancer control vs. Breast cancer group receiving chemotherapy	Breast cancer group receiving chemotherapy vs. Breast cancer group not receiving chemotherapy
	Noncancer control	Breast cancer group not receiving chemotherapy	Breast cancer group receiving chemotherapy			
	Regression coefficient (standard error)					
Pro-inflammatory						
IL-1β	− 0.60 (1.04)	−1.19 (0.82)	1.19 (1.14)	1.19 (1.60)	−1.25 (2.02)	2.43 (1.56)
IL-2	− 0.24 (0.43)	−1.20 (0.55)*	0.70 (0.66)	1.19 (0.67)	−0.44 (0.96)	1.63 (0.89)
IL-6	−0.07 (0.29)	−0.31 (0.37)	− 0.17 (0.26)	0.20 (0.52)	− 0.02 (0.56)	0.22 (0.46)
IL-12p70	−0.005 (0.29)	−0.14 (0.39)	0.81 (0.35)*	0.40 (0.46)	−0.60 (0.60)	0.99 (0.55)
IL-17A	−0.11 (0.17)	−0.15 (0.19)	0.05 (0.22)	0.19 (0.23)	−0.05 (0.33)	0.24 (0.30)
IFNγ	−0.03 (0.08)	−0.07 (0.12)	0.05 (0.14)	0.12 (0.13)	−0.10 (0.19)	0.22 (0.18)
TNFα	0.04 (0.18)	0.02 (0.22)	0.05 (0.20)	−0.10 (0.31)	−0.18 (0.38)	0.07 (0.32)
Anti-inflammatory						
IL-4	−0.01 (0.06)	0.07 (0.06)	0.12 (0.07)	−0.02 (0.09)	−0.06 (0.13)	0.04 (0.11)
IL-5	−0.63 (0.43)	−0.97 (0.52)	0.66 (0.53)	0.65 (0.70)	−0.94 (0.89)	1.59 (0.78)*
IL-10	−0.14 (0.12)	−0.06 (0.14)	0.04 (0.15)	0.05 (0.19)	−0.03 (0.25)	0.08 (0.23)
IL-13	−0.12 (0.19)	−0.29 (0.24)	− 0.04 (0.13)	0.26 (0.32)	− 0.05 (0.31)	0.31 (0.24)

The general linear model is adjusted for age, marital status, and years of education

*IL* interleukin, *IFN* interferon, *Th* T-helper, *TNF* tumor necrosis factor

*p < 0.05

Finally, a similar GLM with adjustment for sociodemographics was established in the groups of patients with breast cancer who were receiving chemotherapy to clarify whether some breast cancer-related factors (tumor mass, duration of completion of chemotherapy, and time since diagnosis of breast cancer) affect cytokine levels. We found that tumor mass and time since diagnosis of breast cancer were not associated with the levels of any cytokine; however, the duration of completion of chemotherapy was associated with IL-5 levels (regression coefficient = 0.009; *P* = 0.043).

## Discussion

To the best of our knowledge, this study is the first to delineate the modified relationships of cancer and chemotherapy between a variety of proinflammatory cytokines and anti-inflammatory cytokines and depression severity in women with breast cancer and controls without cancer. We found that the associations between proinflammatory and anti-inflammatory cytokines and depression severity consistently differed between the groups, especially with regard to IL-5. The differences in IL-5 levels and depression symptoms between groups may explain the development of breast cancer; this finding is also supported by the findings of the association between the duration of completion of chemotherapy and cytokine levels.

We observed differential relationships between IL-5 levels and depression in patients with breast cancer who were receiving and not receiving chemotherapy. Limited information is available in the literature regarding the role of IL-5 in cancer-related depression. Elevated circulatory cortisol levels have been hypothesized to lead to abnormal patterns of synthesis and secretion of IL-5 cytokines, thus provoking the depressive symptoms [26]. Meanwhile, some studies have reported that IL-5 levels can modify the invasion of cancer or the development of metastasis in breast cancer [27] and bladder cancer [28].

The differences in cytokine levels between the groups in our study may be attributed to the development of and change in immune responses during the breast cancer course and chemotherapy treatment, and may also be explained by the theory of immunosuppression, which states that breast cancer cells reduce immune function to escape cytotoxicity from immune cells through the programmed cell death protein 1 (PD-1) pathway [29, 30]. Studies have demonstrated immunostimulation during the early stages of cancer and immunosuppression that occurs after treatment [31, 32]. Although we did not observe higher levels of cytokines in patients with breast cancer compared with controls in our study, depending on the cancer stage of the patients [33], the dynamic change in cytokine levels may have increased the difficulty in capturing such differences in our cross-sectional study design.

Nevertheless, lower inflammation was observed in patients with breast cancer who were receiving chemotherapy, thereby supporting the effect of cancer treatment on the immune system. In addition, the trajectory of depression in our participants is consistent with the findings in the literature. In our study, patients with breast cancer had more severe depression than controls, and patients with breast cancer who were not receiving chemotherapy had the most severe depression. This result is concordant with that of a previous study that pointed out that patients are at the highest risk for depression in the first year after diagnosis of breast cancer [34], and that the symptoms of depression peak immediately after chemotherapy but gradually decrease within half a year after completion of cancer treatment [18].

Our findings suggested that cancer status and cancer treatment can modify the relationships between cytokines and depression severity. Several possible explanations can account for the modification effect. Our findings showed differential relationships of cytokines in depression in the noncancer group and breast cancer groups receiving or not receiving chemotherapy, suggesting that the human body elicits different responses to acute and chronic stress. Such an explanation is supported by the “general adaptation syndrome (GAS)” proposed by Hans Selye to describe a three-stage process (alarm, resistance, and exhaustion) for physiological changes in the human body under long-term exposure to stress, [35] which has been reported to be involved in changes in inflammatory cytokines [36]. Among the three successive stages in chronic stress (GAS), the first (alarm) stage indicates that the individuals are aware of a distress signal and ready to respond to the stressor. The second (resistance) stage indicates that individuals address the stress sustained, and the human body tries to counteract the physiological changes occurring during the alarm reaction stage. The final (exhaustion) stage indicates that after persistently living in a high-stress environment, individuals finally exhaust their energy. These stages might partially reflect the course and treatment of cancer in our groups.

Cytokines respond to acute, sustained, and chronic stress through different mechanisms. In response to acute stress, activation of the hypothalamic–pituitary–adrenal (HPA) axis, sympathetic–adrenal–medullary axis, and vagus fiber promotes the secretions of glucocorticoids, catecholamines, and acetylcholine, respectively, which, in turn, regulate cytokine secretion [36]. Moreover, cytokine responses are not elicited immediately after exposure to an acute stressor, because an increase in the cytokine concentration depends on its production from activated macrophages, endothelial cells, and lymphocytes [37]. Meanwhile, in the event of sustained stress, some changes regulate inflammatory cytokines through HPA “fatigue,” glucocorticoid resistance, inflammation-related transcription pathway activation, and the organism’s negative feedback [36]. Additionally, immunosuppression [38] and cytokine balance [39] are considered defense mechanisms that are activated in response to sustained stress. Finally, if the sustained stress is not removed, the continued increase in inflammatory cytokines leads to inflammation, which causes various diseases [36]. Such dysregulation of cytokines owing to long-term exposure to chronic stress is considered to be the cause of psychiatric diseases [40] and depression [41].

Another possible explanation for the differential relationship of cytokines in depression between patients with breast cancer receiving chemotherapy and those not receiving chemotherapy is that chemotherapy profoundly affects the human body, thereby altering the interaction between cytokines and the biological system. Several findings may support this notion. The administration of chemotherapy agents has been suggested to initiate a series of biological changes—with short-lived alterations in the cytokine milieu inducing persistent epigenetic alterations—that lead to changes in gene expression and alterations in metabolic activity and neuronal transmission [42], all of which play a crucial role in depression [43]. Specifically, it is reported that chemotherapy agents release 5-hydroxytryptamine (HT) from enterochromaffin cells to activate 5-HT3 receptors [44], which are related to depression and are a probable neuronal antidepressant drug target [45]. Further, differential responsiveness to depressive symptoms was observed between patients with advanced cancer who were undergoing chemotherapy and medically healthy patients with depression [46]. These findings indicate that chemotherapy may modify depression through distinct mechanisms, and a deeper understanding of these modifications and mechanisms may help improve the prevention and treatment strategies for depression in patients with cancer.

### Clinical implications

Our findings provide several clinical implications for depression. First, we found differential relationships between cytokines and depression based on cancer status and treatment, indicating that cytokines are essential in the assessment and treatment of depression in patients with cancer both before and after cancer treatment. If cytokines are used as cancer therapy in patients with breast cancer, clinicians should consider the possible development of depressive symptoms after chemotherapy.

### Limitations

Our study has several limitations. First, we used three participant groups to capture the effects of cytokines on depression during the transition from noncancer status to cancer course and cancer treatment. However, owing to the cross-sectional study design, the causality is limited in our study findings. In addition, weak or significant links between cytokine levels and depression severity were merely observed in our study; the small sample size of each study group resulted in limited statistical power and nonsignificant results. Additional longitudinal studies with larger sample sizes are warranted. Nonetheless, previous studies on inflammation resulting from cytokines and depression have been conducted on similarly small samples [15, 18]. Finally, although we included sociodemographic confounders such as age, marital status, and years of education, several confounders such as the use of anti-inflammatory agents were not considered in this analysis.

### Future direction

Several potential mechanisms warrant more studies in future, such as specific differential immunosuppression, acute and chronic stress-related responses in different cytokines, and inflammatory activity between cytokines and enzyme indoleamine 2,3-dioxygenase, kynurenine, and tryptophan, which may be implicated in the cytokine-mediated pathogenesis of cancer and depression.

## Conclusions

The findings of this study reveal the differential relationships between proinflammatory and anti-inflammatory cytokines and depression severity in patients with breast cancer and controls without cancer. No significant associations were observed in the controls. Inverse associations were observed between cytokine levels and depression severity in patients with breast cancer who were not receiving chemotherapy, whereas positive associations were observed in patients with breast cancer who were receiving chemotherapy, after adjustment for sociodemographics.

## Data Availability

The datasets used and/or analyzed during this study are available from the corresponding author on reasonable request.

## Abbreviations

IL: Interleukin
IFN: Interferon
TGF: Transforming growth factor
TNF: Tumor necrosis factor
Th: T helper (cells)
CV: Coefficient of variation
PHQ: Patient Health Questionnaire
GLM: General linear model
GAS: General adaptation syndrome
HPA: Hypothalamic–pituitary–adrenal (axis)
HT: Hydroxytryptamine
